# Evaluation of Load and Stress Distribution for a Novel Design of Maxillary Protraction Facemask by Finite Element Analysis

**DOI:** 10.3390/jcm14082676

**Published:** 2025-04-14

**Authors:** Ghassan Bahir Abdulkareem, Martyn T. Cobourne, Mushriq Abid

**Affiliations:** 1 Department of Orthodontics, College of Dentistry, Al-Bayan University, Baghdad 10070, Iraq; ghassan.bahir@albayan.edu.iq; 2 Centre for Craniofacial Development and Regeneration, Faculty of Dentistry, Oral and Craniofacial Sciences, King’s College London, Guy’s Hospital, Floor 27, London SE1 9RT, UK; martyn.cobourne@kcl.ac.uk; 3 Department of Orthodontics, Faculty of Dentistry, Oral and Craniofacial Sciences, King’s College London, Floor 21, Guy’s Hospital, Guy’s and St Thomas NHS Foundation Trust, London SE1 9RT, UK; 4 Department of Orthodontics, College of Dentistry, University of Baghdad, Baghdad 10070, Iraq

**Keywords:** skeletal Class III, protraction facemask, finite element analysis, 3D scanner

## Abstract

**Background/Objectives:** Protraction facemasks are commonly used to treat Class III malocclusion in growing patients. Personalized facemasks designed using 3D modeling software and based on individual 3D face images are now available. This study aimed to assess the mechanical properties of three novel designs of Petit-type facemask appliances through three-dimensional Finite Element Analysis (FEA). **Methods**: Three novel designs of the facemask were modeled by Solidworks 3D CAD (2023): anatomic, V-shape, and arc-shape. FEA was performed by Ansys 2021 (R2) software. The elements’ sizes, shapes, and numbers were identified, and the material property was set on Acrylonitrile butadiene styrene copolymer (ABS) plastic. The support and loading conditions of two different intensities of load, 7.8 and 9.8 N, respectively, were applied in three angulations to the occlusal plane: 0°, 30°, and 50°. Stress, strain, and total deformation results were obtained. **Results**: The minimum stress was reported with the anatomic design at a 30° angulation, whereas the maximum value was reported in the arc-shape design at 50°; however, there was no significant difference among the three designs. The von Mises yield criterion showed that the overall stresses were distributed on the larger areas of the facemask structure at 30° angulation for all designs. The stresses induced in all facemask appliance designs did not cause permanent deformation. **Conclusions**: Anatomic design has better mechanical behavour than the V-shape or arc shape design. Downward inclination of 30° to the occlusal plane induces less stress. These findings support the use of customized anatomic facemasks for the effective and efficient treatment of Class III malocclusions in growing patients, potentially improving clinical outcomes and patient comfort. Further research, particularly clinical trials, is needed to validate the results of the present study.

## 1. Introduction

Class III malocclusion is considered one of the most difficult discrepancies to treat orthodontically. It has an incidence ranging from 1–4% in Caucasians [1,2]. Orthopedic solutions for Class III malocclusion are seemingly more effective when undertaken at a developmental stage when the jaw sutures are not fully formed. This timing aims to maximize the skeletal effects of treatment while reducing the need for and the complexity of potential future surgical procedures [3,4,5]. This method aims to encourage forward maxillary growth, restrict mandibular growth, and prevent relapses through long-term retention [4,6,7,8,9].

Protraction of the maxillary complex with a facemask is the most widely utilized method in the orthopedic treatment of Class III malocclusion of young adolescent patients [10,11,12,13,14,15]. The facemask components can be adjusted to adapt the appliance exclusively to the patient’s face. This orthopedic protraction typically requires 3.9 to 4.9 newtons (N) of force per side, with an inclination of 30° downward to the occlusal plane [16,17,18,19].

The introduction of fully customized facemask appliances has been made possible by the acquisition of three-dimensional (3D) image data of the patient’s facial anatomy using non-invasive facial planning [20,21,22]. By utilizing these innovative technologies, a Petit-type maxillary protraction facemask with rests, a midline bar, and an elastic attachment bar customized to the patient’s facial anatomy as a single unit can be produced and clinically utilized for a child in need of early treatment for Class III malocclusion [23].

The whole customized facemask structure can be fabricated from a variety of plastic materials, including polyester (PLA), acrylonitrile butadiene styrene copolymer (ABS) polycarbonate (PC), polyetheretherketone (PEEK), and polyacrylate (acrylic, PMMA), which are already widely used in medical devices [24]. In particular for the styrene terpolymer chemical family, ABS is the most common carbon chain copolymer. ABS plastic is a well-known material for its applications in different industries including the medical field [24,25,26,27]. The high tensile strength, stiffness, high impact resistance, and toughness of ABS polymer are valuable properties for mechanical applications [28,29].

Facemask component manipulation plays a vital role in controlling load application during maxillary protraction therapy [18]. Load magnitude, direction, and duration may produce several patterns of forces distribution and maxillary complex displacement [16,17,18]. Although many studies have investigated stress and load distribution on the facial complex during maxillary protraction by means of finite element analysis (FEA) [30,31,32], only one study has evaluated the mechanical properties of the Delaire facemask [14]. Regarding the fully customized Petit-type facemask, there are no data available in the literature investigating its mechanical properties of its innovative design. Though the relevance of customized 3D-printed appliances is rising, biomechanical research on their performances in relation to conventional designs still lags behind. Although several studies have looked at how well 3D-printed appliances work, only a few have systematically assessed their load and stress distributions by utilizing computer modeling tools [14,33]. The present study offers a facemask design that is fully customized to a particular patient and could be fabricated from polymer material in a single unit, matching patient demands with no modification throughout the treatment interval. The main proposed novelty of the current facemask design lies in its optimized force application, which is meant to enhance maxillary protraction while minimizing undesirable side effects such as dental tipping or soft tissue irritation. The design’s included structural modifications and material options are meant to improve patient comfort and the appliance’s functionality. However, earlier studies have not adequately examined how these elements affect the general efficacy of 3D-printed appliances. Finite element analysis introduces a powerful tool for the evaluation of the biomechanical properties of orthodontic and orthopedic appliances. Unlike clinical trials or physical models, FEA offers a thorough examination of force transmission, stress distribution, and structural reliability under controlled conditions. This work offers insights into the potential benefits of the new facemask design in clinical applications by using FEA to compare it with current designs. Hence, the aim of the present study was to evaluate deformation and stress distribution on three assorted designs of the Petit-type facemask by means of FEA. The goal was to identify the optimal design that produces clinically acceptable levels of deformation and stress distribution under varying force magnitudes and directions. The present study may be considered as a contribution to the field by its systematic evaluation of the mechanical performance of a novel 3D-printed facemask, focusing on force vectors, stress distribution, and structural stability. By addressing the limitations of conventional designs and leveraging computational modeling, this research seeks to provide valuable insights into the clinical applicability of customized facemask therapy for Class III malocclusion treatment.

## 2. Material and Methods

The FEA requires identification of the following parameters:

1. Geometrical features of the facemask, constructed by 3D modelling software (SOLIDWORKS (2023). Corp., Waltham, MA, USA);
2. Material properties for all elements of the facemask, ABS;
3. Meshing properties (sizes, shapes, and numbers of the elements used to discretize the facemask);
4. Constraints and loads conditions applied on the system.

**Figure 1:**
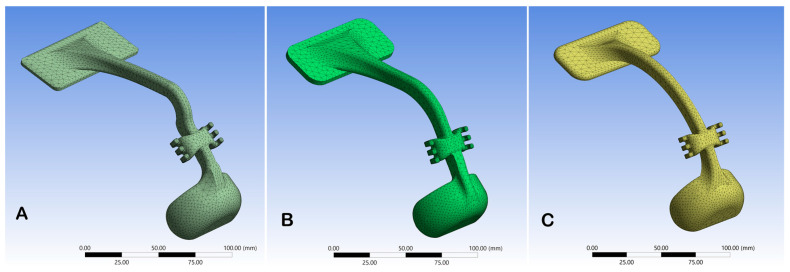
Three-dimensional model meshes of (**A**) anatomic design with 36,859 nodes and 21,061 elements, (**B**) V-shape design with 39,985 nodes and 23,105 elements, and (**C**) arc-shape design with 38,603 nodes and 22,146 elements, characterized by the numerical model.

Three-dimensional imaging of the face for a young patient (11 years old) with Class III malocclusion was obtained using the Revopoint Pop 3D scanner (Revopoint 3D Technologies Inc., Shenzhen, China). The patient was asked to sit in a resting position, while the 3D scanner rotated around the head as needed for proper image capturing. The acquired 3D image was exported in STL.file format, and three facemask designs were constructed by Solidworks 3D Computer Assisted Design (CAD) software. An anatomic design following the patient’s facial anatomy, a V-shape design morphology, and an arc-shape design were used (Figure 1).

**Figure 2:**
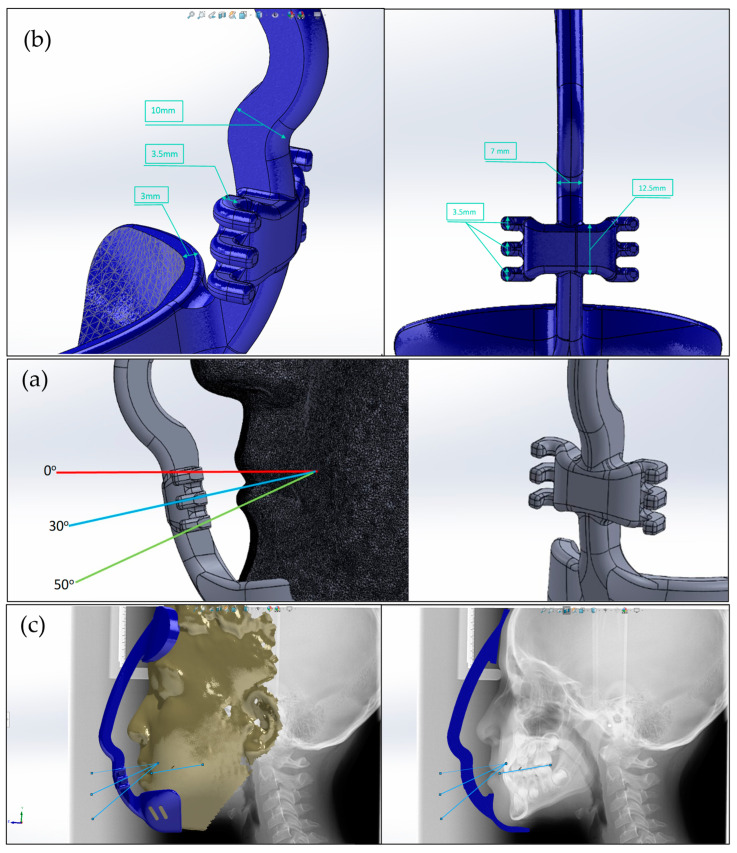
(**a**) Geometric dimensions of facemask design. (**b**) Design of the horizontal arm of the customized facemask. (**c**) Integration of cephalography into Solidworks for accurate angulation to the occlusal plane.

**Figure 3:**
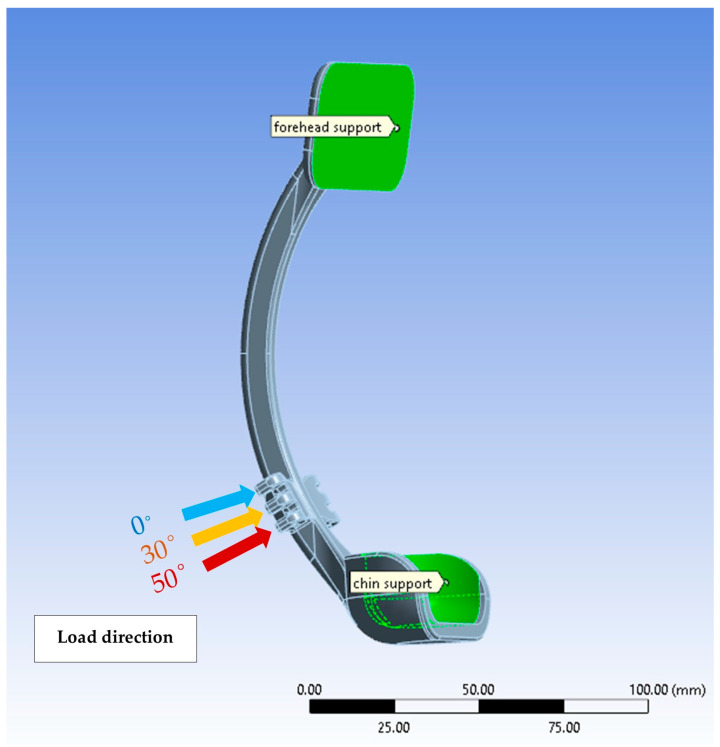
Schematic representation of boundary conditions and load application points and directions in relation to the occlusal plane.

The design’s geometric dimensions are shown in Figure 2a. All the designs were composed of a single vertical arm connecting a forehead and chin rest pads and a horizontal arm at the level of the resting lip line (Figure 2b). The horizontal arm of the facemask was designed to fit the inter-commissural width of the patient mouth, with three projections for elastic attachments, representing the angulation of the force to the occlusal plane. Incorporating lateral cephalography into Solidworks software enabled accurate angulation to the occlusal plane (Figure 2c). Boundary conditions and load application points and directions in relation to the occlusal plane are illustrated in Figure 3: the upper projection is 0°, the middle one is 30°, and the lower projection is 50°.

## 3. Ansys Simulations

The Ansys 2021 (R2) static structural simulation was used to perform the FEA. Nonlinear analysis was used in this study, which is more accurate for ABS plastic material. For the meshing process of the simulation software, the elements’ sizes and shapes were set by software preference according to the geometry of the design. Tetrahedral (Tet) elements with 10 nodes (quadratic tet) were used by accomplishing adaptive meshing with gradual element-size transitions to prevent numerical errors. Tetrahedral elements can easily adapt to any shape and produce better accuracy for complex and irregular geometries that have stress concentration zones necessitating a finer mesh. A mesh convergence study was conducted to verify mesh independence, using three mesh densities (coarse, medium, and fine). The variation in maximum von Mises stress between the two finest meshes was below 5%, indicating that the results had converged and were not significantly affected by further mesh refinement. For accurate simulation of ABS in an FEA environment, we input the materials’ properties. The physical properties of the ABS material used in the simulation process are listed in Supplemental Table S1. Validation ABS material for the FEA model was made by the simulation of a tensile test in accordance with ASTM D638 standards [34]. The simulation results were benchmarked against experimental data from a study by Tymrak et al., which reported an average tensile strength of 28.5 MPa and an elastic modulus of 1807 MPa for ABS specimens produced by fused deposition modeling (FDM) [35]. Focusing on elastic modulus, yield strength, and ultimate tensile strength, the stress–strain response of our FEA model, compared with these experimental results, facilitates the elimination of computational and experimental data discrepancies. This validation approach improved the reliability of the FEA estimates, warranting that the behavior of the simulated material was closely matched the actual mechanical performance of ABS.

A static simulation of FEA was performed for the evaluation process of customized facemask behavior by the application of a static load in two different intensities: 7.8 and 9.8 N. These static loads were applied in three different inclinations to the occlusal plane (0°, 30°, and 50°) to highlight the relationship among the loads, constraints, and characteristics of the facemask materials. Loads were applied at the elastic projections of the facemask bilaterally; the upper projection received force at a 0° angle to the occlusal plane, the middle projection received force at a 30° angle, and the lower projection received force at a 50° angle. Fixed supports were defined at the forehead and chin (Figure 3). Von Mises yield criterion was used to visually evaluate all the loads’ conditions and constraints and to highlight the displacement and stress distribution on the facemasks’ structure.

## 4. Results

The designs’ geometric dimensions, elements, and node numbers derived from the meshing process are shown in Supplemental Table S2. The realized numerical model for each design is shown in Figure 1. A mesh convergence study was conducted to ensure that the results were independent of mesh density. Quadratic tetrahedral (10-node) elements were used with adaptive meshing. Two levels of mesh refinement were assessed as follows: coarse mesh: 31,026 nodes and 19,742 elements and fine mesh: 78,752 nodes and 48,291 elements. The maximum principal stress changed from 3.0927 MPa to 3.1599 MPa, with a relative change of 2.151%, which is within the predefined 20% convergence criterion. This confirms that further mesh refinement would have a negligible impact on the results.

**Figure 4:**
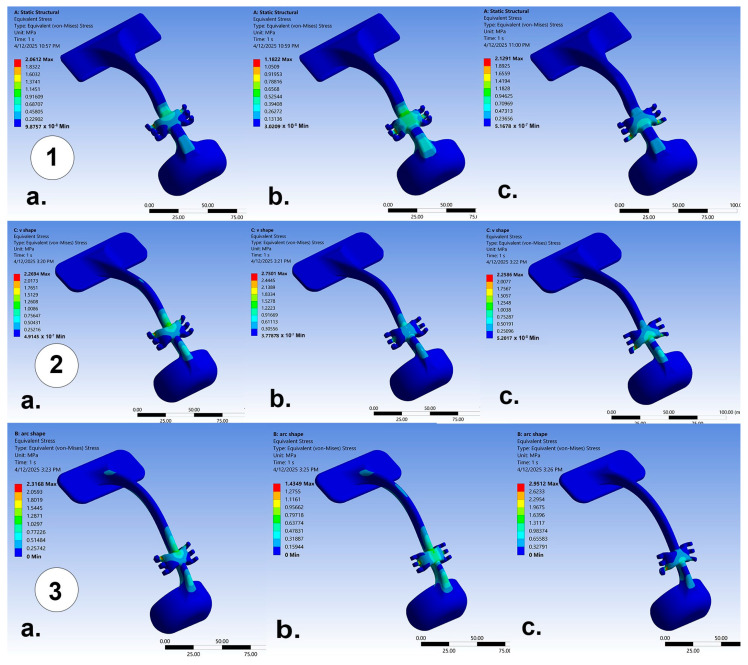
Distribution of stress with a traction load of 9.8 newtons applied on the anatomical (**1**), V-shape (**2**), and arc-shape designs (**3**). The force direction was progressively inclined to the occlusal planes of (**a**) 0°, (**b**) 30°, and (**c**) 50°.

The results of maximum stress, maximum strain, and total deformation for the three different designs (anatomic, V-shape, and arc-shape) under different load intensities (7.8 N and 9.8 N) and angulations to the occlusal plane (0°, 30°, and 50°) are shown in Table 1, Table 2, and Table 3, respectively. The resulting stress and strain were reported in newtons per square millimeter (N/mm^2^) and millimeter per millimeter (mm/mm), respectively. As illustrated by Table 1, the lowest stress was (0.97 N/mm^2^) reported with the anatomic design at 30° angulation to the occlusal plane and a 7.8 N load intensity, whereas the highest value was (3.11 N/mm^2^), reported in the arc-shape design at 50° angulation with a 9.8 N load intensity. Additionally, the anatomic design showed relatively constant stress across different angulations, with slight increases in stress at higher loads (1.63 N/mm^2^), whereas higher stress was reported for the V-shape and arc-shape designs as compared with the anatomic design, especially at 50° angulation. Regarding maximum strain and total deformation, the analysis showed similar findings (Table 2 and Table 3). The anatomic design showed relatively low strain compared with the other designs, ranging from 3.84 × 10^−4^ to 6.77 × 10^−4^ mm/mm. The highest strain was reported in the arc-shape design, especially at higher loads (12.07 × 10^−4^ mm/mm at 9.8 N and 50° angulation), whereas the V-shape design showed a moderate level of strain ranging from 6.12 × 10^−4^ to 9.39 × 10^−4^ mm/mm. The von Mises yield criterion is shown in Figure 4, represented by a color-coded illustration of the stress distribution of the three designs across different angulations of the applied forces, where red/yellow indicates high stress concentration, while green/blue indicates low stress concentration. Figure 4 highlights the fact that the stress patterns in the von Mises yield criterion align with the values in Table 1, and the stress concentration appears mainly around the central horizontal component (load application area), suggesting that this region experiences the highest forces. It also suggests that the stress intensity of all the designs increases with angulation. An overall insight of the results data is that the anatomic design has the lowest stress, strain, and deformation levels, making it the most structurally stable and durable among the three in different loading conditions.

## 5. Discussion

An orthopedic facemask appliance is the recommended treatment for growing Class III patients presenting with maxillary deficiencies [4,6,7,8]. Several studies have been conducted that aim to enhance the fitness and comfort of facemasks [36,37,38]; however, there is no fully customized facemask described in the literature. This study aimed to investigate three innovative designs of fully personalized facemasks fabricated from ABS plastic, followed by FEA to assess the stress and strain distributions across these designs under various loading conditions. FEA is a widely used technique for the analysis of the stress and load distribution induced on skeletal structures during the protraction of the maxilla [32,39,40]. This method involves the simulation of mechanical forces from a situation with infinite elements in which the real one is compared to a simulation with finite elements. Internal stress and strain can therefore be obtained, which are essential and whose direct measurement would be difficult to obtain if adequate computational models were not available.

A previous study analyzed a facemask structure to calculate stress and strain distribution by FEA and reported that the overall stresses generated in the facemask structure were below the elastic limit (yield point) of the applied material [14]. Here, we report that there were no risks of permanent plastic deformation for all three designs of facemask structures. For the different load conditions, FEA showed that the maximum generated stress in all the tested designs at 9.8 N of force were inferior to the ultimate tensile strength of the ABS material (Table 1). All three designs presented greater tensile strength values than the resulting stresses in the simulation process. The maximum stress reported on the facemask structure at the lower horizontal projection (50° angulation to the occlusal plane) of the arc-shape design was 3.11 N/mm^2^, while the tensile strength of the ABS material was equal to 43 N/mm^2^. The lowest stress concentration was observed for the anatomic design at an angulation of 30° to the occlusal plane for the 7.8 N load intensity, which is consistent with the findings of a previous study [14]. The von Mises yield criterion showed that the overall stresses were distributed on larger areas of the facemask structure at 30° angulation for all designs (Figure 4).

The maximum strain values of all three designs (Table 2) showed a strain range from 3.84 × 10^−4^ (mm/mm) for the anatomic design at 30° with a load intensity of 7.8 N to 12.07 × 10^−4^ mm/mm for the arc-shape design at 50° with a 9.8 N load, which were not significantly different and would not cause a structural concern, given that the Young’s modulus of ABS material is 2390 mega pascal (MPa).

During orthopedic treatment of Class III malocclusion, careful facemask management plays a vital role in optimizing performance. The structures of the new fully customized facemask appliance, the horizontal arm position, and the chin and frontal support regions need to be individually adjusted to fit the patient’s face to provide a more homogeneous distribution of the applied loads. Most studies confirmed that, to induce more efficient anterior displacement of the maxilla, force magnitudes should range from 7.8 to 9.8 N [16,19,41]. According to previous findings, the most favorable force vector is represented by a 30° inclination to the occlusal plane [16,17,19]. Hence, it allows a favorable maxillary-complex rotation in a counterclockwise direction and reduces stress and tensile forces on the facemask device, as shown by our results. Force vector inclination is firmly related to the vertical position of the horizontal bar. Therefore, it is necessary to calibrate the force vector direction at 30° by incorporating a cephalometric inclination of the occlusal plane during the modeling process of the facemask. Although all the tested designs exhibit a minute amount of elastic strain and deformation, our study showed that all the tested designs did not demonstrate any permanent changes when force levels of 7.8–9.8 N were applied, eliminating any risk of load dispersion. However, the anatomic design showed a minimum amount of stress, elastic strain, and total deformation, which may indicate a better choice for the clinical application of fully customized facemasks in the early treatment of skeletal class-III malocclusion. Our study investigated the mechanical response of the face mask by the FEA method; however, the biomechanical reaction of the alveolar bone and the skin of resting places on the face may be explored in a future study.

## 6. Clinical Implications of the Study

The findings of the present study offer an important clinical insight for the treatment of skeletal Class-III malocclusion with orthopedic maxillary protraction. Results of finite element analysis (FEA) show how changes in facemask (FM) design, particularly the direction and angulation of the force vector, can greatly affect the stress distribution and displacement pattern in the craniofacial bone. This means that facemask selection should be personalized according to the specific anatomical and developmental characteristics of the patient. For instance, a patient with a more retrusive maxilla may benefit from a more downward and forward force vector, as this orientation promotes anterior and inferior maxillary displacement and potentially improves the orthopedic results. Careful modulation of the force vector is required for patients with vertical growth tendencies or excessive mandibular to avoid clockwise rotation of the mandibles. Nevertheless, regardless of the precise vector, the highly adaptive sutures of younger patients may respond more favorably to facemask forces, whereas older teenagers may benefit from a design modification that concentrates higher stress at the crucial sutural areas to maximize skeletal response. By providing evidence-based guidance on how different FM force vectors interact with craniofacial structures, this study supports a more tailored approach to appliance design and force application for improving treatment outcome and patient comfort while minimizing unwanted side effects.

## 7. Strength and Limitations

The present study provides valuable insight into the mechanical behavior of unique designs of facemasks used in the interceptive treatment of Class III malocclusion. However, some limitations exist in relation to the accuracy of the FEA results, which are dependent on the accuracy of the models created by Solidworks and Ansys software. These models may simplify the complex biomechanics involved in the interaction between the facemask and the patient’s facial structure. Another limitation is that anatomical variations existing across different patients, such as bone density, sutural maturation, or craniofacial morphology, could influence stress distribution and deformation outcomes and may not be captured by a single patient model. Future research incorporating a wider range of patient models with different ages, sexes, and malocclusion severities may be helpful to validate and expand on these results clinically in a more diverse context.

## 8. Conclusions

This study reinforces the superiority of the anatomic design in terms of mechanical behavior and provides insight into optimal traction angles. This study suggests practical implications by affirming the suitability of ABS plastic for facemask fabrication and providing guidance on design considerations for better treatment outcomes. Additionally, modifications in facemask force direction during the treatment of Class III malocclusion have a significant influence on the pattern of stress distribution and maxillary displacement, and the 30° downward force vector was most effective in producing anterior and inferior movement of the maxilla, along with preserving the structural balance of the facemask appliance. These findings recommend the customization of facemask design according to the anatomical and developmental characteristics of every patient. The present study provides valuable biomechanical evidence to support more informed clinical decision-making in maxillary protraction therapy.

## Figures and Tables

**Table 1:** Maximum stress (N/mm^2^) of the anatomic, V-shape, and arc-shape designs with loads of 7.8 N and 9.8 N in 0°, 30°, and 50° angulation to the occlusal plane, respectively.

Load Intensity (N)	7.8			9.8		
Angulations to Occlusal Plane	0	30	50	0	30	50
Anatomic	1.27	0.97	1.29	1.6	1.22	1.63
V-shape	1.83	1.21	1.84	2.30	1.52	2.31
Arc-shape	1.87	1.11	2.48	2.35	1.38	3.11

**Table 2:** Maximum strain (mm/mm) of the anatomic, V-shape, and arc-shape designs, with loads of 7.8 N and 9.8 N in 0°, 30°, and 50° angulation to the occlusal plane, respectively.

Load Intensity (N)	7.8			9.8		
Angulations to Occlusal Plane	0	30	50	0	30	50
Anatomic	5.23 × 10^−4^	3.84 × 10^−4^	5.39 × 10^−4^	6.57 × 10^−4^	4.83 × 10^−4^	6.77 × 10^−4^
V-shape	7.47 × 10^−4^	6.12 × 10^−4^	7.45 × 10^−4^	9.39 × 10^−4^	7.69 × 10^−4^	9.36 × 10^−4^
Arc-shape	7.59 × 10^−4^	4.56 × 10^−4^	9.61 × 10^−4^	9.54 × 10^−4^	5.70 × 10^−4^	12.07 × 10^−4^

**Table 3:** Total deformation (mm) of the anatomic, V-shape, and arc-shape designs, with loads of 7.8 N and 9.8 N in 0°, 30°, and 50° angulation to the occlusal plane, respectively.

Load Intensity (N)	7.8			9.8		
Angulations to Occlusal Plane	0	30	50	0	30	50
Anatomic	2.15 × 10^−2^	1.53 × 10^−2^	1.37 × 10^−2^	2.70 × 10^−2^	1.93 × 10^−2^	1.73 × 10^−2^
V-shape	2.99 × 10^−2^	1.77 × 10^−2^	2.09 × 10^−2^	3.76 × 10^−2^	2.23 × 10^−2^	2.63 × 10^−2^
Arc-shape	3.16 × 10^−2^	1.95 × 10^−2^	2.35 × 10^−2^	3.97 × 10^−2^	2.38 × 10^−2^	2.96 × 10^−2^

## Data Availability

The materials and results of this study belong to the authors and are only available upon request, after agreement by the author.

## Supplementary Materials

The following supporting information can be downloaded at: https://www.mdpi.com/article/10.3390/jcm14082676/s1.
