# Supplementary material for: Evaluation of Load and Stress Distribution for a Novel Design of Maxillary Protraction Facemask by Finite Element Analysis
Source: J Clin Med. 2025 Apr 14;14(8):2676. doi: 10.3390/jcm14082676 (PMC12028103; doi:10.3390/jcm14082676)
Supplement: Supplementary file 1 [file jcm-14-02676-s001.zip › jcm-3542057-supplementary.pdf]

Supplemental tables

Supplemental Table S1. Mechanical properties of the ABS material.

Young's Modulus	2390 MPa
Poisson's Ratio	0.399
Bulk Modulus	3943.9 MPa
Shear Modulus	854.18 MPa
Tensile Ultimate Strength	43 MPa
ensile Yield Strength	41.4 MPa

Supplemental Table S2. 3D design dimensions, element and nodes number for anatomical, V-shape, and arc-shape design.

Design	Length X mm	Length Z mm	Length Z mm	Nodes	Elements No.
Anatomic	82.55	172.02	79.601	36859	21061
V-shape	82.55	171.52	72.877	39985	23105
Arc-shape	82.55	170.66	68.915	38603	22146
